# Improving Consumer Understanding of Medical Text: Development and Validation of a New SubSimplify Algorithm to Automatically Generate Term Explanations in English and Spanish

**DOI:** 10.2196/10779

**Published:** 2018-08-02

**Authors:** Nicholas Kloehn, Gondy Leroy, David Kauchak, Yang Gu, Sonia Colina, Nicole P Yuan, Debra Revere

**Affiliations:** ^1^ Department of Linguistics University of Arizona Tucson, AZ United States; ^2^ Computer Science Department Pomona College Claremont, CA United States; ^3^ Department of Spanish and Portuguese University of Arizona Tucson, AZ United States; ^4^ Health Promotion Sciences Division Mel and Enid Zuckerman College of Public Health University of Arizona Tucson, AZ United States; ^5^ Department of Health Services School of Public Health University of Washington Seattle, WA United States

**Keywords:** text simplification, health literacy, natural language processing, terminology

## Abstract

**Background:**

While health literacy is important for people to maintain good health and manage diseases, medical educational texts are often written beyond the reading level of the average individual. To mitigate this disconnect, text simplification research provides methods to increase readability and, therefore, comprehension. One method of text simplification is to isolate particularly difficult terms within a document and replace them with easier synonyms (lexical simplification) or an explanation in plain language (semantic simplification). Unfortunately, existing dictionaries are seldom complete, and consequently, resources for many difficult terms are unavailable. This is the case for English and Spanish resources.

**Objective:**

Our objective was to automatically generate explanations for difficult terms in both English and Spanish when they are not covered by existing resources. The system we present combines existing resources for explanation generation using a novel algorithm (SubSimplify) to create additional explanations.

**Methods:**

SubSimplify uses word-level parsing techniques and specialized medical affix dictionaries to identify the morphological units of a term and then source their definitions. While the underlying resources are different, SubSimplify applies the same principles in both languages. To evaluate our approach, we used term familiarity to identify difficult terms in English and Spanish and then generated explanations for them. For each language, we extracted 400 difficult terms from two different article types (General and Medical topics) balanced for frequency. For English terms, we compared SubSimplify’s explanation with the explanations from the Consumer Health Vocabulary, WordNet Synonyms and Summaries, as well as Word Embedding Vector (WEV) synonyms. For Spanish terms, we compared the explanation to WordNet Summaries and WEV Embedding synonyms. We evaluated quality, coverage, and usefulness for the simplification provided for each term. Quality is the average score from two subject experts on a 1-4 Likert scale (two per language) for the synonyms or explanations provided by the source. Coverage is the number of terms for which a source could provide an explanation. Usefulness is the same expert score, however, with a 0 assigned when no explanations or synonyms were available for a term.

**Results:**

SubSimplify resulted in quality scores of 1.64 for English (*P*<.001) and 1.49 for Spanish (*P*<.001), which were lower than those of existing resources (Consumer Health Vocabulary [CHV]=2.81). However, in coverage, SubSimplify outperforms all existing written resources, increasing the coverage from 53.0% to 80.5% in English and from 20.8% to 90.8% in Spanish (*P*<.001). This result means that the usefulness score of SubSimplify (1.32; *P*<.001) is greater than that of most existing resources (eg, CHV=0.169).

**Conclusions:**

Our approach is intended as an additional resource to existing, manually created resources. It greatly increases the number of difficult terms for which an easier alternative can be made available, resulting in greater actual usefulness.

## Introduction

### Background and Significance

Text is an important resource for health-related information as it is easy to create and distribute. Furthermore, health literature is widely available in the form of web-based resources for people to obtain information on medical conditions, diseases, and modalities [1]. However, these documents are often written at a level beyond the comprehension of the average reader [2]. This disconnect reflects an overall trend in misinformation regarding health conditions [3,4].

To mitigate this problem, researchers have sought automatic ways to improve the readability of these texts and the resulting reader comprehension. This natural language programming (NLP) task is known as *text simplification* [5] and has been used to create supervised [6], semisupervized [7], and fully automatic tools [8] to make texts easier for consumers to digest by increasing readability [9]. A central challenge for this research is to develop resources and techniques that enhance the quality and accuracy of these systems. Even though deep neural network approaches and other automated translation algorithms are increasingly being developed, it will take time before they can be applied with sufficient impact and precise simplifications. We intend for our algorithm to supplement existing resources as well as generate useful inputs for other algorithms.

The first step is identifying what makes text difficult. Some of the previous studies have focused on simplifying individual terms, while others have focused on grammatical structures. To identify the difficulty of individual terms, we use *term familiarity*. For a given term, this measure can be calculated by extracting the likelihood that a term occurs in common language usage [10], which we estimate according to the term’s frequency in the Google Web Corpus [11]. In this work, we add to the body of research that identifies these terms and replaces them with easier synonyms [12]. However, we went beyond existing approaches by generating new explanations for terms that do not exist in the available resources. To do this, we developed and evaluated a new algorithm to generate new explanations. We generated explanations of terms in plain language using word internal parsing and affix dictionaries with SubSimplify.

### Resources for Finding Explanations for Difficult Terms

Ideally, there would be an endless resource of expert-written explanations for difficult terms, optimized for the general public in multiple languages. However, few resources are able to provide appropriate explanations at all and even fewer are able to automatically or semiautomatically produce such explanations.

The resource closest to ideal is the English Consumer Health Vocabulary (CHV) [13], which is included in the Unified Medical Language System (UMLS) [14]. This resource was manually created and provides synonyms as well as definitions for medical terms in a consumer-friendly language. For the purposes of text simplification, these plain language definitions and simple synonyms double as ready-made explanations for difficult terms. However, the number of explanations is low relative to the overall number of difficult terms that occur in a given medical text. The CHV contains 2567 unique definitions and 88,529 synonyms for concepts found in the UMLS. We did not employ the UMLS as a resource because this system focuses on mapping complex medical concepts onto ontologies and is not designed to relate health information to patients or any other person outside the medical domain.

Previous research has shown that the CHV can be used to simplify texts [15-17], but it has also been shown to contain jargon words and not enough consumer-friendly vocabulary when providing summaries for specialized research [18]. Furthermore, while this resource is well tailored to text simplification, it is limited to English terms and explanations. In summary, the CHV provides explanations that can be automatically sourced in a given simplification system. However, CHV is only in English, is for relatively few terms, and can at times contain jargon beyond the reading level of the average reader.

While not being medically focused, WordNet is a useful resource for text simplification. It is an online lexical database containing terms and definitions, as well as interword semantic relations such as hypernyms, hyponyms, synonyms, and antonyms [19]. WordNet provides 128,391 word-sense definitions in English and is also available in Spanish, albeit in a less complete form [20]. Since WordNet is not a medical resource, many of its explanations are not optimal for medical text simplification, and when several senses are provided for a word, it is not always clear which best suits the medical sense. Previously, WordNet has been used to provide synonyms for lexical simplification [21]. For example, hyponym-hypernym relations have been used to generate synonyms that are simpler (more general) for text simplification [22]. In other areas, this resource has been used to simplify texts in the domain of biomolecules [23] and in texts written for non-native English speakers [24]. In summary, while WordNet is larger than CHV and also available in Spanish, the resource is not always optimal for giving the definition for medical terms.

Recent developments using neural networks trained on large bodies of text have produced larger resources such as word embeddings, where words are represented by multidimensional word vectors. The resulting vectors position the word relative to each other in a multidimensional space and have been shown to possess semantic and syntactic relations that allow us to automatically find synonyms and semantically related terms [12]. Given a word, we can use its vector representation to find the word whose vector is nearest to this word. Often, this nearest vector is a synonymous word. One freely available version of this resource is the pretrained Global Vectors for Word Representation (also known as GLoVe) [25]. Prior work has shown that these vectors can prove to be useful to isolate simple yet more frequent terms in the areas of text simplification [26]. However, they can include spurious matches because the approach cannot differentiate antonyms from synonyms. Given that this resource is totally automated, a word vector model can be produced from any language given a relatively large body of text. This means that this resource is also available for Spanish, with pretrained vectors available online [27]. In our study, we employed the GLoVe pretrained vectors for English and for Spanish [27], labeling the approach more generally as Word Embedding Vectors (WEV).

In all, the methods that exist for explanation generation range from specific, and precise, with low coverage to high coverage, with a much lower relative accuracy. In the next section, we describe our approach, which exists on the spectrum between these resources.

## Methods

### Using Morphological Information to Generate Explanations

We first describe the role that morphological units play in medical terminology and then our algorithm, which extracts information and generates explanations using these morphological units.

The resources described above make use of a word’s definition in isolation without reference to the internal characteristics of that word, (ie, the morphology of the word). While it is not always the case, often romance languages contain morphological units that contain relatively clear semantics, such as the case for the prefix *anti*- (“against”), or the suffix -*s* (indicating plural). In certain words in English and in Spanish, these can help one to decipher the meaning of a word. In medicine, many terms, both in English and Spanish, originate from Greek and Latin [28,29]. Greek and Latin affixes have meanings commonly unknown to the average reader, but they nevertheless reflect the overall meaning of a word. While at times the meaning of a word is a direct function of the composition of the meaning of these morphological units, to a large degree in English and Spanish, terms composed of these units tend to have a gestalt effect. On the extreme end, a term may completely differ from the meaning of its morphological units (eg, “ledger” does not mean “ledge”+*er*). However, this problem of semantic drift is small for medical terms, seemingly because medical terms are less affected by semantic drift than more nonmedical, frequent terms.

Affixes that compose medical terms commonly have clear definitions that reflect a word’s meaning. For example, given the prefix *cardio-* we know that this term’s meaning relates to “the heart.” Several resources containing these affixes and their definitions are freely available online [30-33]. From these, we created a unique dictionary of affixes along with their definitions for each language. We extracted 586 unique affixes for English and 498 affixes for Spanish. We define an affix as any morphological unit that has some denotation apart from the word itself. Affixes are categorized by their position, with prefixes occurring at the beginning of the words and suffixes occurring at the end of words. A root is any morphological unit that can stand alone as a single word. For example, the term *cardiovascular* contains the prefix *cardio-*, and the root *vascular*. Independently, these morphological units denote *the heart* and *consisting of a vessel or vessels*, respectively. Although many resources may not contain a definition for *cardiovascular*, by parsing these morphological units, we can automatically generate an explanation that reflects the actual denotation of the term: *relating to the heart and blood vessels*.

In both Spanish and English, words may be composed of multiple suffixes, roots, and prefixes. SubSimplify exploits this fact to generate an explanation for a term. Table 1 shows the examples of affixes and their definitions in both Spanish and English.

In addition to these affix dictionaries, we use word stemming [34] to isolate stemmed, or lemmatized, versions of terms. Stemming and lemmatization are two different methods of reducing a term to something similar to its root, but in a way that does not always reflect the actual root. For example, a resource like WordNet may have a definition for *Gastrointestine*, but not *Gastroinstestinal*. By stemming and stripping the affix *-al*, we increase the ability to find explanations using all resources.

**Figure 1:**
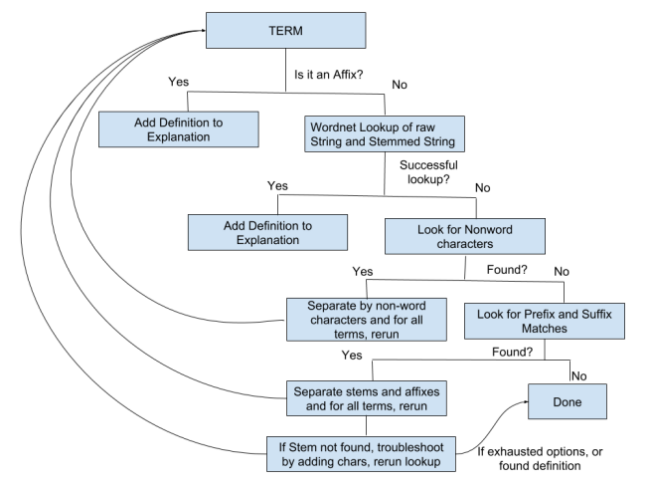
SubSimplify flow diagram.

Figure 1 provides an overview of our SubSimplify algorithm. The input to SubSimplify is a term we assume to be difficult, and we recursively lookup affixes and generate an explanation by accumulating the definitions of each affix and root identified. When finished, we align these definitions to provide an explanation of the term.

We use affix dictionaries to identify morphological units programmatically. First, the system identifies affixes and then takes the part of the word that is not an affix and performs a database lookup on stemmed variants of the term. To avoid spurious matches, we work from larger to smaller suffixes, and thus, *anti-* as in *anti-hero* would match before *a-* as in *a-symmetry*. This process occurs iteratively until no affixes are matched, or until there is no root left. In order to describe this process in sequence, Textbox 1 gives a detailed description of each step.

Since words may contain multiple suffixes, the process occurs multiple times where possible. That is, when we extract a root, it is possible that that root may yet contain another suffix or prefix. To highlight this, we provide an example with the term *hyperglycemic* in Textbox 2.

**Table 1:** Examples of affixes and corresponding definitions in English and Spanish.

Language and affix		Type	Definition	Origin
**English**				
	adip-	prefix	*Of or relating to fat or fatty tissue*	Latin
	-dipsia	suffix	*(condition of) thirst*	Greek
**Spanish**				
	pireto-	prefix	*Forma prefija que significa fiebre*	Latin or Greek
	-opsia	suffix	*Forma sufija que significa visión*	Greek

**A description of each step in the SubSimplify algorithm.**

Affix Identification:

All affixes in the affix dictionary are compared to the term from the longest to shortest length. If the term contains the affix characters at the beginning (for prefixes) or ending (for suffixes), the system considers this an affix match.

Affix Definition:

For each affix match, the affix dictionary definition is added to the newly constructed explanation.

Root Extraction:

The root of the term is extracted by removing the prefix or suffix. Since this can remove some of the characters of the root unintentionally, we consider the root the remaining characters plus single character variations of the root at the edge where the term was matched.

Search Resources:

The extracted root is then searched in WordNet and in CHV. If it is not found, we reintroduce the root to this same process until no matches are found.

**SubSimplify algorithm application to hyperglycemic.**

Affix Identification:

We iteratively go through the affix dictionary and match the prefix *hyper-* in *hyperglycemic*.

Affix Definition:

The definition for *hyper* —“*denotes something as extreme or beyond normal”* —is added to the explanation for the term *hyperglycemic*.

Root Extraction:

We extract *glycemic* from *hyperglycemic.*

Search Resources:

WordNet and CHV are searched for *glycemic* and all single character variants of *glycemic* (eg, *aglycemic*). When not found, we rerun this entire process on *glycemic*, saving the explanation so far.

**Table 2:** Example English explanations.

Explanation resource	Example term	Explanation
CHV^a^	*pheochromocytoma*	A usually benign, well-encapsulated, lobular, vascular tumor of chromaffin tissue of the Adrenal Medulla
WordNet Summary	*Coryza*	an inflammation of the mucous membrane lining the nose (usually associated with nasal discharge)
WordNet Synonym	*attenuated*	rarefy
SubSimplify	*hyperglycemic*	hyper-glyc-em-ic, “extreme” or “beyond normal”-sugar-em-pertaining to
Word Vector Nearest Neighbor	*toxoplasma*	gondii

^a^CHV: Consumer Health Vocabulary.

This process repeats until there is either no root left, or until the remaining root fails to be identified by any resource. For *glycemic*, the system will identify *-ic* and subsequently *glyc-* before halting at *-em-*.

If the term contains “-” or any other *nonword* characters, we split these as well. The parsed affixes and roots are then aligned with their explanations to provide an affix-by-affix breakdown of the term. For any affix that is not identified in the system, as is the case with *-em-* in *hyperglycemic*, the definition of the root remains the root itself. Upon presenting the term, these affixes are matched with their definition both by order and by color in order to make identification as easy as possible for a writer. An example explanation for *hyperglycemic* is shown in Table 2. This table contains explanations for a few different difficult terms to highlight their quality when present. Note that not all resources contain explanations for all terms, so it is extremely rare that all resources can provide an explanation for a single term.

While the CHV [35] and WordNet Summary resources provided full-sentence explanations (semantic simplification), the WEV and WordNet Synonym provide single-word explanations of each term (lexical simplification). SubSimplify provides a hybrid of the two: for the individual parsed subword units, either a synonym or brief description is presented.

Next, we describe 2 studies designed to evaluate the quality, coverage, and usefulness of these explanations in English and Spanish.

### Studies

To evaluate the quality, coverage, and usefulness of the newly generated explanations and how they compare to existing resources, we conducted two studies: one in English and one in Spanish.

#### Study 1: English Term Explanation Generation

##### Study Stimuli

###### Stimuli

To obtain a range of medical terms that occur in common texts, we extracted 20 documents from Wikipedia written on a medical topic and 100 PubMed abstracts. From these documents, we extracted the difficult terms using term familiarity. For the purposes of this study, we identified difficult terms as those having a frequency less than the 5000th ranked term in the Google Web Corpus, which previous work showed to be a reasonable criterion [7]. Given these difficult terms, we selected 200 terms from each resource type (PubMed and Wikipedia) balanced across all documents (100 and 20, respectively). To investigate the effect of frequency, we also balanced each set of 200 difficult terms by frequency. Two groups were extracted based upon high and low frequencies. High-frequency terms were those which had frequencies in the upper most tertile, and low-frequency terms were those which had frequencies in the lowest tertile. In all, the study contained 400 total terms that were evenly split across high and low frequency, document source, and the documents themselves.

###### Explanation Generation

We compared our approach to four previous approaches: CHV, WordNet Synonyms and Summaries, and WEV. These resources provided explanations when an exact match could be found for the term in their database.

##### Metrics

For each of the 400 terms, we calculated 3 metrics: quality, coverage, and usefulness. Quality was judged by subject experts (SEs). The SEs in this study were required to (1) be a native speaker of the language and (2) have at least a master’s degree in a public health or a medical-related field. The experts typically had experience evaluating the quality of medical resources, and for this study, they were financially compensated for their time.

For quality*,* the two SEs reviewed each term along with the candidate definitions and explanations. For each definition or explanation, the SEs annotated how useful it was on a 4-point Likert scale. Table 3 provides a description of each rating level. Coverage was measured by calculating the percentage of terms for which an explanation was provided by each source. Usefulness is a broader measure than quality and takes the availability of terms and resources into account. When a term is not found, it receives a score of 0. While quality gives us an idea of how accurate resource explanations are, usefulness tells us how well such a resource would perform if we were to employ it for all terms.

##### Procedure

The SEs evaluated the 400 terms and the corresponding explanations provided by each resource. The order of the presentation of explanations was randomized for each term. For each of the terms, the SEs scored the term on both the quality and coverage metrics described above. We then calculated usefulness by normalizing quality by coverage.

**Figure 2:**
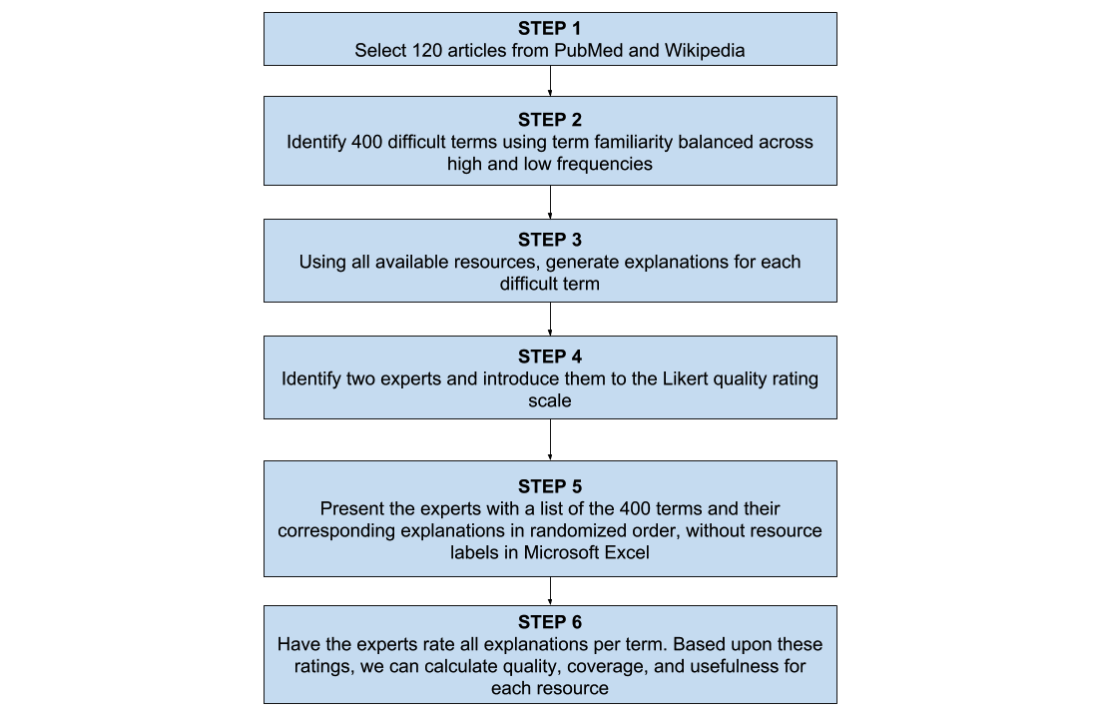
Steps for English term explanation generation study.

In order to give a visual idea of how this study was performed, Figure 2 contains a flowchart containing the steps of the study.

**Table 3:** Likert quality scale.

Rating	Description
1	Explanation *is not useful* to someone annotating the text.
2	Explanation *is a little useful* to someone annotating the text.
3	Explanation *is useful* to someone annotating the text.
4	Explanation *is very useful* to someone annotating the text.

**Table 4:** English study results.

Metrics	CHV^a^	WordNet Synonym	WordNet Summary	SubSimplify	WEV^b^
Quality (1-4 scale)	2.81	2.09	3.32	1.64	1.64
Coverage (N=400), %	6.0	53.0	53.0	80.5	83.8
Usefulness (0-4 scale)	0.169	1.11	1.76	1.32	1.38

^a^CHV: Consumer Health Vocabulary.

^b^WEV: Word Embedding Vector.

##### Evaluation Outcomes

###### Interoperator Variability

To compare the variability in quality scores between each of the SEs, we calculated Crohnbach alpha. Since we did not limit the quality ratings to a rank order, it was possible for each term to have multiple explanations that received the best score per term. Therefore, we calculated Crohnbach alpha in two ways. First, in a conservative version, we calculated whether each SE chose all of the same explanations as the best for each term, and in a more liberal version, whether each SE chose one of the same explanations as the best for each term. For English, the results were 0.69 and 0.90 for the conservative and liberal version, respectively. We, therefore, determined that interoperator reliability was high enough to average their ratings. Table 4 shows the results of the quality, coverage, and usefulness metrics for each explanation source in English.

In Table 4, we see that each column represents explanation sources and the 3 rows give the metrics averaged across SEs. For example, CHV received a mean quality score of 2.81 when present, but could only provide explanations for 24 out of 400 total terms. Subsequently, its usefulness was only 0.169 for the 400 terms. Recall that this resource represents the one that is manually generated to aid lexical simplification in medical documents. As a consequence, the quality rating was relatively high, but the coverage was by far the lowest. Next, we see that WordNet Summaries and Synonyms each provided the same number of explanations. However, the Summaries (Semantic Simplification) scored much higher than the Synonyms (Lexical Simplification) at 3.32 versus 2.09, respectively. Again, given that they only provide explanations for 212 terms, their usefulness was only 1.76 and 1.11, respectively. While SubSimplify had a 1.64 quality score when present, its coverage was 322, whereas that of WordNet was 212, representing an increase from 53.0% to 80.5% in coverage of the 400 *difficult terms*. Consequently, the usefulness of SubSimplify was 1.32, greater than that of WordNet Synonyms and CHV. Last, WEV provided the greatest coverage and performed identically to SubSimplify in quality (1.64), but had greater coverage (335) and quality (1.38). However, as we describe in the next subsection, there was a clear difference between the quality performance of SubSimplify and WEV. SubSimplify performed better with lower-frequency words and in more technical literature than WEV.

###### Quality

To evaluate significance, we performed a 2×2×5 analysis of variance (ANOVA), with quality as the dependent variable. The independent measures were document source (Wikipedia or PubMed), frequency (Low or High), and the five explanation sources (CHV, WordNet Synonym, WordNet Summary, SubSimplify, and WEV). There were main effects for frequency (*F*_1,2186_ = 3.859, *P*<.02) and explanation type (*F*_4,2186_ = 260.1, *P*<.001). This indicates that on average, the resources performed significantly better with lower-frequency terms and that there were significant differences between the resources.

**Figure 3:**
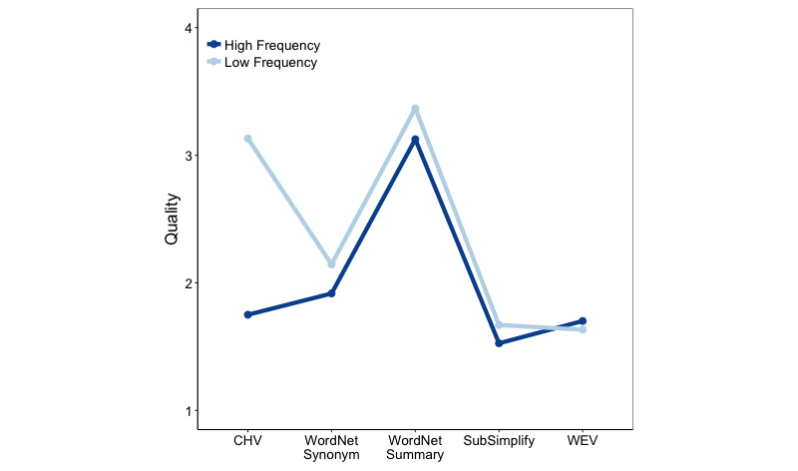
Explanation type-frequency interaction for quality in English. CHV: Consumer Health Vocabulary, WEV: Word Embedding Vector.

In addition to the main effects, there was a significant two-way interaction between explanation type and frequency (*F*_8,2186_=2.993, *P*<.001; Figure 3). Figure 3 contains the mean quality of each resource at low and high frequencies. Given that our documents contained medical terminology, we expected low-frequency words to be the rarest and, therefore, most technical. They presented the hardest target for any system attempting to summarize these documents. For example, CHV, which is written specifically for medical terms, has much greater performance for low-frequency terms than for high-frequency terms (3.12 vs 1.74). Furthermore, WordNet Synonyms and Summaries both performed slightly better for low-frequency terms than for high-frequency ones. Interestingly, SubSimplify also followed this pattern. However, WEV had the opposite trend. Not only did WEV perform better on high-frequency terms than on low-frequency terms but also it performed slightly poorer than SubSimplify for low-frequency terms (compare SubSimplify’s 1.67 rating to WEV’s 1.63 rating for low-frequency terms).

###### Coverage

To evaluate the effect of frequency and document source on the coverage of each resource, we performed another 2×2×5 ANOVA with coverage as the dependent variable. There were main effects for frequency (*F*_1,3970_=3.859, *P*<.001) and explanation type (*F*_4,3970_=260.1, *P*<.001; refer to Table 2, row 2). This indicates that explanations, on average, had significantly greater coverage for high-frequency terms than for low-frequency terms.

**Figure 4:**
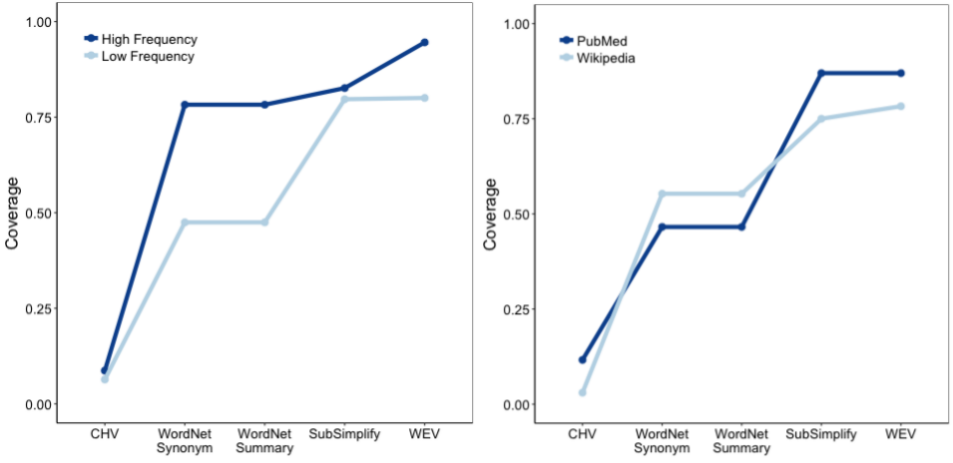
Coverage-frequency interaction (left) and coverage-document type interaction (right) in English. CHV: Consumer Health Vocabulary, WEV: Word Embedding Vector.

There was a significant two-way interaction between explanation type and frequency (*F*_8,3970_=6.557, *P*<.001) and a significant interaction between explanation type and document source (*F*_4,3970_=11.523, *P*<.001; Figure 4).

As seen in the left side of Figure 4, term frequency affects the coverage of each explanation type. Whereas CHV had similar coverage for low- and high-frequency terms, WordNet had much greater coverage for high-frequency terms than for low-frequency terms (0.47 vs 0.77). SubSimplify then increased the coverage of low-frequency terms from 0.47 to 0.78 and of high-frequency terms from 0.77 to 0.79. Last, WEV slightly increased the coverage of low-frequency terms (from 0.77 to 0.78), but increased that of high-frequency terms from 0.79 to 0.94. This indicates that SubSimplify performed quite similar on low- and high-frequency terms, as is the case with CHV, but with an overall much greater coverage.

Next, we look at the interaction of document source and coverage. Recall that PubMed contains more technical medical terms than Wikipedia sources, and therefore, it constitutes terms that should contain more technical jargon. In the right side of Figure 4, we see that CHV, SubSimplify, and WEV each had greater coverage in PubMed than in Wikipedia, whereas WordNet had greater coverage in Wikipedia documents. The x-axis depicts the proportional coverage for each explanation source in PubMed and Wikipedia, and the y-axis includes the change in coverage. WordNet, for example, provides fewer explanations for PubMed (0.48) than for Wikipedia (0.52), whereas SubSimplify provides more for PubMed (0.8) than for Wikipedia (0.75). One critical point to note is that SubSimplify has equivalent coverage to WEV. This indicates that SubSimplify performed as well as the fully automated WEV within technical medical text.

###### Usefulness

Next, we performed another 2×2×5 ANOVA with usefulness as the dependent variable. There was only a main effect for explanation type (*F*_4,3970_=95.170, *P*<.001; refer to Table 2, row 3) and frequency (*F*_1,3970_=14.663, *P*<.001). This indicates that the usefulness ratings were significantly different across the different explanations and that usefulness ratings were significantly greater for high-frequency terms on average.

**Figure 5:**
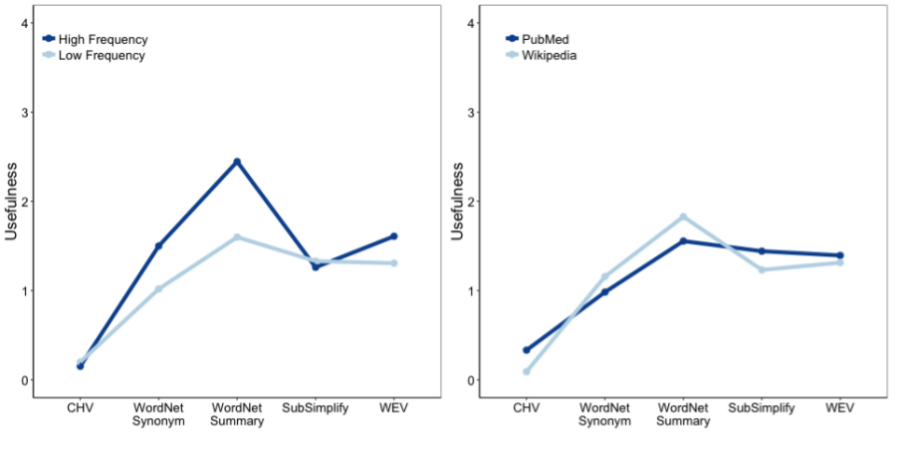
Explanation type-frequency interaction (left) and explanation type-document source interaction (right) in English usefulness measures. CHV: Consumer Health Vocabulary, WEV: Word Embedding Vector.

There was a significant two-way interaction between explanation type and frequency (*F*_7,3970_=5.390, *P*<.001) and a significant interaction between explanation type and document source (*F*_4,3970_=6.387, *P*<.001; Figure 5).

As seen in Figure 5 on the left, term frequency affected the usefulness of each explanation type. While CHV had a greater usefulness for low-frequency versus high-frequency terms, WordNet Synonyms had much greater usefulness for high-frequency than low-frequency terms (1.50 vs 1.00). WordNet Summaries had the greatest usefulness score for high-frequency terms (2.40) and smaller, but still quite high, scores for low-frequency terms (1.62). Meanwhile, SubSimplify had greater usefulness for low-frequency terms than for high-frequency terms (1.43 vs 1.35). Last, WEV had a greater score for high-frequency than for low-frequency terms (1.64 vs 1.42). This affirmed the idea that SubSimplify is a resource that performs best for low-frequency terms.

Next, we look at the interaction of document source and coverage. Recall that PubMed contains more technical medical terms than Wikipedia sources, and, therefore, constitutes terms that should contain more technical jargon. In Figure 5, we see that WordNet performed better in Wikipedia and that SubSimplify and CHV both performed better in usefulness for PubMed. This affirmed that SubSimplify performs best on more technical documents.

##### Summary

For the English study, we found that SubSimplify performed better than existing medical resources for coverage and had a relatively high quality given its coverage. Furthermore, SubSimplify, much like CHV, performed better for low-frequency and more technical terms, than for high-frequency terms. In addition, this resource had the greatest coverage of all the resources for terms found in the PubMed abstracts. These quality, coverage, and usefulness results suggest that SubSimplify is better equipped to generate explanations for low-frequency and technical terms than the other existing resources.

#### Study 2: Spanish Term Explanation Generation

The second study evaluated our approach in Spanish. This study was identical to the English study, save for two differences. First, there was no Spanish language CHV and WordNet in Spanish only contained summaries (no Spanish synonyms; the version we used contained only Spanish terms mapped to English synonyms). Therefore, we only used WordNet Summaries and compared only three possible explanation resources: our approach (SubSimplify), WordNet, and WEV. Second, since there were no Spanish language PubMed abstracts available, for our second resource, we used Medline Plus [36] instead, which is a resource for medical articles geared toward people interested in health information. Last, all instructions, ratings, and explanations were in Spanish.

##### Study Stimuli

###### Stimuli

We tested 400 medical terms balanced for frequency. To get a range of medical terms that occurred in both common texts and more technical texts, we extracted 20 documents from Wikipedia written on the topic of disease and 20 Medline Plus articles. From these documents, we first extracted difficult terms using the same term familiarity threshold. Our cutoff was a frequency less than the 5000th most common term in the Spanish Google Web Corpus [10]. Within these terms, we balanced the terms across low and high frequency. In all, we split these 400 terms across high and low frequency and document source.

###### Explanation Generation

We compared our approach to two previous approaches: WordNet and WEV. These resources provided explanations when an exact match could be found for the term in their database.

##### Metrics

For each of the 400 terms, we extracted explanations. For each explanation, we calculated quality, coverage, and usefulness.

##### Procedure

The procedure was identical to English except that all instructions and explanations were written in Spanish. The SEs were both bilingual Spanish-English speakers who had nevertheless received Master of Public Health degrees in English.

##### Evaluation Outcomes

###### Interoperator Variability

Again, we calculated Crohnbach alpha in both a liberal and conservative version. For Spanish, the results were 0.64 and 0.90 for the conservative and liberal versions, respectively. We, therefore, again determined that the interoperator reliability was high enough to collapse their quality ratings into one group.

Table 5 shows the results of the quality, coverage, and usefulness for each explanation source in Spanish. It can be seen that WordNet had the highest average quality rating of the three resources (2.64), but provided the lowest coverage at 20.5%, with a low resulting usefulness (0.543). The coverage of SubSimplify was much greater at 90%, with a lower average quality rating (1.49). Last, WEV provided a higher quality rating (1.84) but with a lower coverage than SubSimplify (89.75%). Regarding usefulness, WEV outperformed SubSimplify (1.77 vs 1.24).

Given these results, we performed ANOVAs to understand the relationship between frequency and document source for the quality, coverage, and usefulness of each explanation type.

###### Quality

We performed a 2×2×3 ANOVA to evaluate the effect of document source (Wikipedia or PubMed) and frequency (High or Low) on the quality ratings for each of the three explanation resources (WordNet, SubSimplify, and WEV). There were main effects for frequency (*F*_1,1590_=13.39, *P*<.001) and explanation type (*F*_2,1590_=98.805, *P*<.001; refer to Table 3 row 1). This indicates that on average, the explanations performed significantly better on high-frequency terms and that there was a significant difference between the average quality of explanations based upon their type.

**Figure 6:**
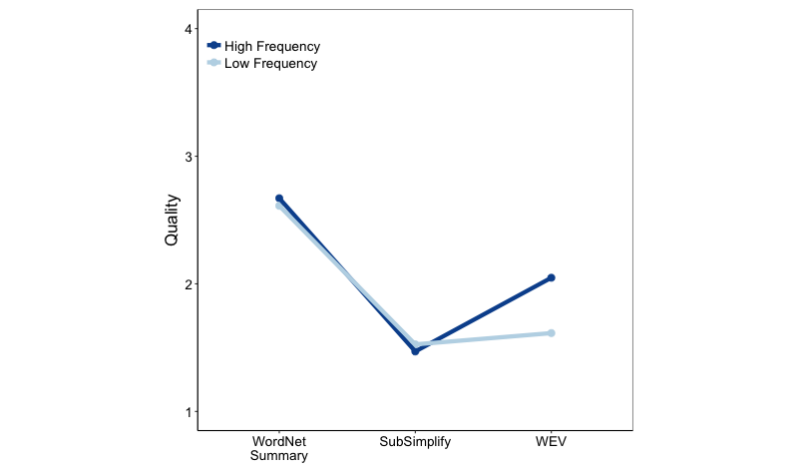
Explanation type-frequency interaction for quality in Spanish. WEV: Word Embedding Vector.

There was also a significant two-way interaction between explanation type and frequency (*F*_2,1590_=12.010, *P*<.001; Figure 6). Figure 6 shows the interaction of frequency with explanation type. The x-axis depicts the mean quality for each explanation source at low and high frequency, and the y-axis includes the change in mean quality ratings as a line. SubSimplify performed better for low-frequency terms (1.54) than for high-frequency terms (1.48), whereas WEV performed worse for low-frequency terms (1.68) than for high-frequency terms (2.03).

###### Coverage

We performed a 2×2×3 ANOVA to evaluate the effect of document source (Wikipedia or MedlinePlus) and frequency (High or Low) on the coverage ratings for each of the three explanation resources (WordNet, SubSimplify, and WEV). There were also main effects for frequency (*F*_1,2382_=7.180, *P*<.001) and explanation type (*F*_2,2382_=1142.361, *P*<.001; refer to Table 3). This indicates that on average, the explanations had significantly better coverage on high-frequency terms and that there was a significant difference between the average coverage of explanations based upon their type.

**Figure 7:**
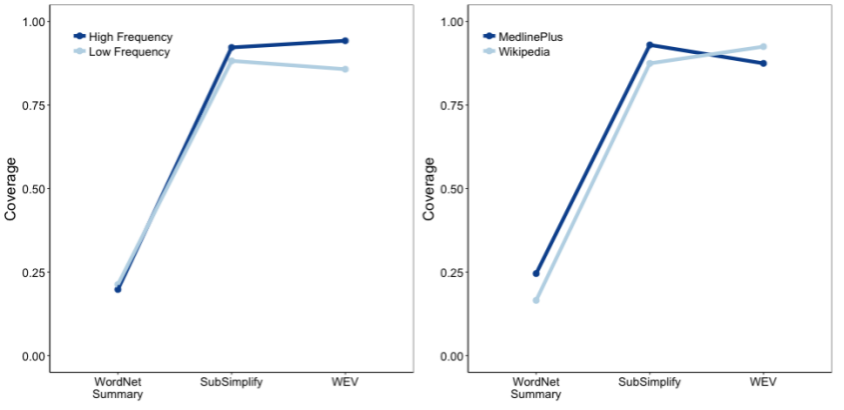
Coverage-frequency interaction (left) and coverage-document type interaction (right) in Spanish. WEV: Word Embedding Vector.

There was a significant two-way interaction between explanation type and frequency (*F*_2,2382_=4.465, *P*<.015), and a significant interaction between explanation type and document source (*F*_4,2382_=6.259, *P*<.001; Figure 7). For Spanish terms, term frequency affected the coverage of each resource as seen in Figure 7 on the left. For WordNet, there was slightly greater coverage for low-frequency terms (0.22) than for high-frequency terms (0.20), but both were quite low. For SubSimplify, there was greater coverage for high-frequency terms (0.92) than for low-frequency terms (0.87). This was also the case for WEV, with high and low coverages at 0.93 and 0.86, respectively. This indicates that SubSimplify had the greatest coverage for low-frequency terms and WEV had the greatest coverage for high-frequency terms in Spanish.

For document source, WordNet had a greater coverage for MedlinePlus terms (0.25) than for Wikipedia terms (0.20). Likewise, SubSimplify performed better on the more technical terms of MedlinePlus (0.93) than on the more general terms of Wikipedia (0.87). WEV, however, had the opposite effect, with 0.93 for Wikipedia and 0.86 for MedlinePlus. In short, WEV performed better on less technical texts and higher-frequency terms, whereas SubSimplify performed better on low-frequency terms and more technical texts.

**Table 5:** Spanish study results.

Metrics	WordNet Summary	SubSimplify	WEV^a^
Quality (1-4 scale)	2.64	1.49	1.84
Coverage (N=400), %	20.5	90.0	89.7
Usefulness (0-4 scale)	0.543	1.24	1.77

^a^WEV: Word Embedding Vector.

**Figure 8:**
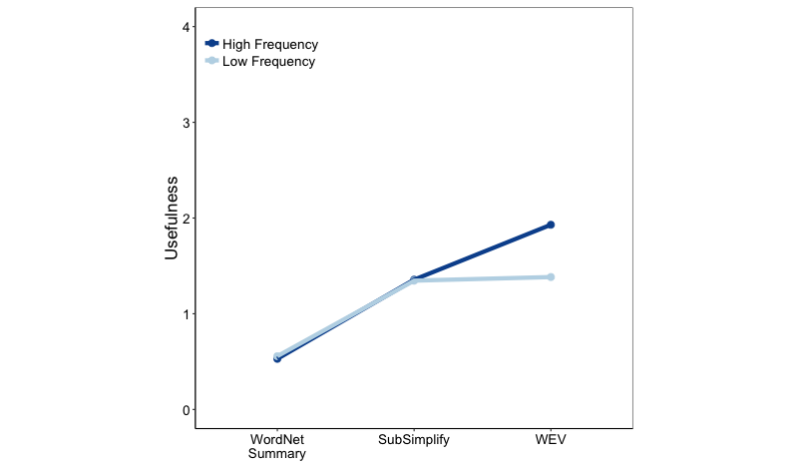
Explanation type-frequency interaction for frequency. WEV: Word Embedding Vector.

###### Usefulness

We performed the same 2×2×3 ANOVA, but this time with usefulness as the dependent measure. The results indicated main effects for frequency (*F*_1,2382_=16.197, *P*<.001), explanation type (*F*_2,2382_ = 230.268, *P*<.001), and document source (*F*_1,2382_=6.737, *P*<.001). These results indicate that explanations, on average, performed significantly better on high-frequency terms and on Wikipedia documents. This also indicates that there was a significant difference in the usefulness measures for explanation type (refer to Table 3, row 3).

Furthermore, there was a significant two-way interaction between explanation type and frequency (*F*_2,2382_=17.911, *P<*.001). Figure 8 depicts this interaction. Notice that WordNet Summaries performed nearly identically on low- and high-frequency terms (0.6). This pattern was also true for SubSimplify (1.4 for both). Last, WEV performed much better in usefulness on high-frequency terms than on low-frequency terms (1.9 vs 1.4).

###### Summary

For the Spanish study, we found that SubSimplify performed better than WordNet and WEV for coverage. Specifically, it performed best for low-frequency and more technical terms; its average quality was lower than that of the other two resources, but was better at low frequency.

## Results

In both English and Spanish, SubSimplify had its best quality ratings at low frequency, which were similar for both languages. Furthermore, in both languages, SubSimplify had similar results regarding coverage. In both the English and Spanish studies, we saw that SubSimplify greatly outperformed the existing resources with its ability to provide multiword explanations for difficult terms. Namely, SubSimplify outperformed CHV and WordNet Summaries in English in quality, and in Spanish, it outperformed WordNet in this same measure. Furthermore, it provided the most explanations at low frequencies and in more technical texts.

At the same time, much of the quality and coverage that we have shown covers overlapping data. Here we have evaluated the coverage of these resources if we were to employ all of them into a single system. Doing so will highlight the role that SubSimplify can play in a larger simplification system. Given the 400 terms in each language, the charts in Figure 7 highlight the cumulative coverage of each resource. Given that not all resources cover the same words, these bar graphs show the coverage of a system that includes each nonoverlapping explanation or synonym from the previous resource.

Left, CHV provides the lowest coverage with 23, WordNet then provides 212, SubSimplify provides 322, and, finally, WEV provides 336 out of 400. On the right, we see the number of explanations that our system can provide as we add each resource in Spanish. For example, if we only used CHV, we would only be able to provide explanations for 23 terms. However, as we add each resource, the number of (nonoverlapping) terms for which we can provide explanations increases. As we add WordNet, we can provide explanations for 222 terms; then by adding SubSimplify, we can provide explanations for 349. Last, by adding WEV, we can provide a total of 385 explanations out of the 400 total terms, or 99% of all terms.

**Figure 9:**
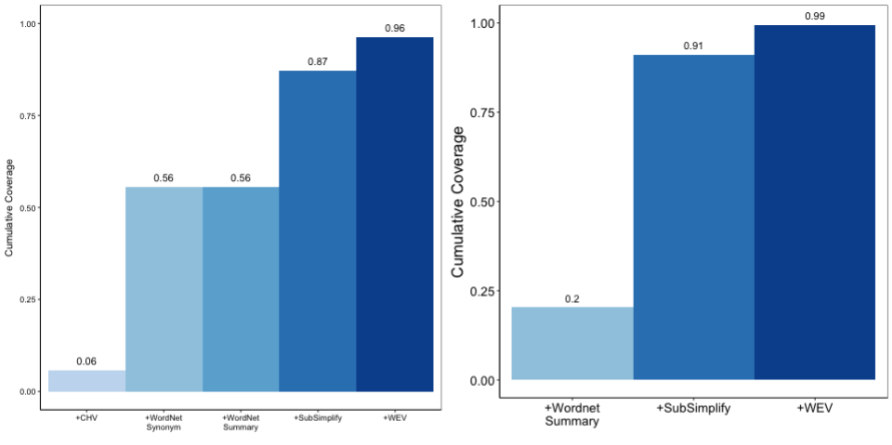
Cumulative coverage in English (left) and Spanish (right). CHV: Consumer Health Vocabulary, WEV: Word Embedding Vector.

**Figure 10:**
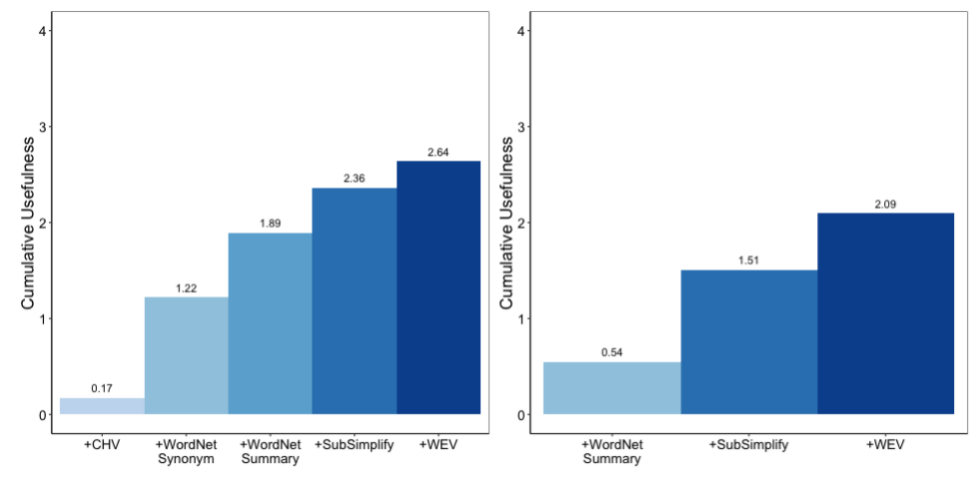
Cumulative usefulness in English (left) and Spanish (right). CHV: Consumer Health Vocabulary, WEV: Word Embedding Vector.

SubSimplify acts as an intermediate resource between the fully automatic, synonym-providing source of WEV and the annotator-written resources of CHV and WordNet. In its semiautomated approach, it increases the coverage of total terms for which any simplification system can provide multiword explanations. This also is made apparent when we look at the cumulative usefulness of all explanations, which can be found in Figure 9. Here we have provided the cumulative quality rating for all terms additively. For example, in English (left), the CHV quality is the average rating of explanations for all 400 terms, most of them being 0. Then +WordNet Synonyms gives the average quality of these two resources combined. When two resources each provide an explanation, we take the higher rated explanation of the two. The result is that by employing SubSimplify in conjunction with all resources, this simplification system can provide explanations with a 2.64 usefulness rating in English and 2.09 in Spanish (right).

## Discussion

### Principal Findings

The aim of the English and Spanish studies was to evaluate the efficacy of employing SubSimplify to medical texts, and the results revealed what was expected. Compared with WordNet and CHV, the quality of explanations was, on average, lower. This may be an indication of a few different issues, which we will expand upon in the Limitations.

Surprisingly, the fully automatic system of WEV outperformed our expectations. In creating SubSimplify, we imagined that there would be many spurious matches and synonyms that were unrelated to the difficult terms, but the results showed a better performance than expected. Based upon this, we are motivated to employ WEV as another way to source root synonyms in SubSimplify. That is, in its current form, SubSimplify performs a term lookup in WordNet (and CHV, in English) after parsing each affix. Based upon these results, we are motivated to have the system perform a WEV lookup at this stage as well.

One challenge with SubSimplify is to maximize the understandability of the explanations themselves. While the existing resources contain a single definition of the term, SubSimplify relies upon combining multiple definitions to sew together a single explanation. Currently, the system employs a color-coding scheme that relates the morphological units on one line to their definitions on another. This may make it difficult to read for some people, effectively lowering the quality when people are not used to seeing these definitions. In order to allay this issue, our team plans on implementing a few different formats and testing them out in the near future in an interactive Web program.

### Limitations

SubSimplify is naturally limited by two factors. First, not all difficult medical terms contain subword units, and additionally, not all subword units match or are totally accurate. This is because not all terms contain morphological units, and the system has no way of knowing where the characters within a word are actual examples of suffixes. Beyond that, even if they are correct matches of affixes, there is no guarantee that the actual meaning of the terms is directly reflective of the affixes that are found within. For example, it may provide a meaning for *anti-* and -*bodies* in *antibodies*. But the explanation *against bodies* does not reflect the actual meaning of the term.

Another possibility is that many of the explanations generated by SubSimplify can be incomplete. For example, it may provide a meaning for -*al* in *distal* but no meaning for *dist-*. The result would then be difficult for anyone to understand. Nevertheless, the system does provide a bridge between hand annotated and automatic texts and, therefore, should be subject to these sorts of exceptions and problematic cases.

Second, SubSimplify, by virtue of using WordNet and CHV, is limited as well to the coverage of those resources. However, we believe that this work presents useful addition to a system aimed at providing explanations for complex terms.

### Conclusions

The niche of SubSimplify is to exploit the regularities of morphological units in medical terminology to provide a window into breaking down the jargon of difficult terms into digestible terms. SubSimplify will improve as the resources used to create it do. Furthermore, we want to look at multiword phrases as oftentimes they reveal the contextual meaning that a single-word context cannot provide alone. This approach is intended as an additional resource that one can add to other methods to automatically provide explanations for difficult texts. The explanations generated by this system greatly increase the number of difficult terms for which an easier alternative can be made available and, thereby, present an advance in the area of text simplification in the medical domain.

## Abbreviations

ANOVA: analysis of variance
CHV: Consumer Health Vocabulary
NLP: Natural Language Processing
SE: subject expert
UMLS: Unified Medical Language System
WEV: Word Embedding Vector
